# Modeling of the GC content of the substituted bases in bacterial core genomes

**DOI:** 10.1186/s12864-018-4984-3

**Published:** 2018-08-06

**Authors:** Jon Bohlin, Vegard Eldholm, Ola Brynildsrud, John H.-O. Petterson, Kristian Alfsnes

**Affiliations:** Norwegian Institute of Public Health, Lovisenberggata 8, P.O. Box 4404, 0403 Oslo, Norway

**Keywords:** Microbial genomics, Core genome, Mathematical modeling, SNP GC content, Core genome GC content, Statistical parameter estimation

## Abstract

**Background:**

The purpose of the present study was to examine the GC content of substituted bases (sbGC) in the core genomes of 35 bacterial species. Each species, or core genome, constituted genomes from at least 10 strains. We also wanted to explore whether sbGC for each strain was associated with the corresponding species’ core genome GC content (cgGC). We present a simple mathematical model that estimates sbGC from cgGC. The model assumes only that the estimated sbGC is a function of cgGC proportional to fixed AT*→*GC (*α*) and GC → AT (*β*) mutation rates. Non-linear regression was used to estimate parameters *α* and *β* from the empirical data described above.

**Results:**

We found that sbGC for each strain showed a non-linear association with the corresponding cgGC with a bias towards higher GC content for most core genomes (66.3% of the strains), assuming as a null-hypothesis that sbGC should be approximately equal to cgGC. The most GC rich core genomes (i.e. approximately %GC > 60), on the other hand, exhibited slightly less GC-biased sbGC than expected. The best fitted regression model indicates that GC → AT mutation rates *β = (1.91 ± 0.13) p < 0.001* are approximately (1.91/0.79) = 2.42 times as high, on average, as AT→GC *α = (− 0.79 ± 0.25) p < 0.001* mutation rates. Whether the observed sbGC GC-bias for all but the most GC-rich prokaryotic species is due to selection, compensating for the GC → AT mutation bias, and/or selective neutral processes is currently debated. Residual standard error was found to be *σ = 0.076* indicating estimated errors of sbGC to be approximately within ±15.2% GC (95% confidence interval) for the strains of all species in the study.

**Conclusion:**

Not only did our mathematical model give reasonable estimates of sbGC it also provides further support to previous observations that mutation rates in prokaryotes exhibit a universal GC → AT bias that appears to be remarkably consistent between taxa.

**Electronic supplementary material:**

The online version of this article (10.1186/s12864-018-4984-3) contains supplementary material, which is available to authorized users.

## Background

GC content in bacterial genomes varies greatly from, for instance, 13.5% in the intracellular symbiont *Candidatus* Zinderia insecticola [1] to more than 75% in the soil dwelling *Anaeromyxobacter dehalogenans* [2]. This variance in base composition has been found to be driven by phylogeny [3], environment [4], selection [5] and selective neutral processes [6, 7] as well as drift due to a general AT mutation bias [8–10]. Indeed, it has been shown that for several intracellular microbes the genomes tend to be small, less than 0.2 M base-pairs (mbp) and often AT rich [1] while soil bacteria have large (several mbp), GC-rich genomes [11]. Population structure, absence or low rates of recombination and dominating AT mutation bias appear to guide the intracellular genomes towards higher AT content [12], presumably due to relaxation of selective constraints [1]. Analogous genomic patterns are also observed for free-living bacteria exposed to similar relaxation of selective pressures [13, 14]. A recent study [15] showed that the core genomes are slightly, but significantly, more GC rich than the corresponding accessory genes, i.e. genes belonging to the less conserved part of the genome in a species. This was assumed to be a consequence of purifying selection operating on the core genomes [5], although selective neutral processes such as GC-biased gene conversion (gBGC) [6] and/or amelioration [7] could not be dismissed [15]. Accessory genes of bacterial species are more frequently distributed between microbes with the possible consequence that purifying selection will not have the opportunity to purge fitness reducing mutations, which are often biased towards higher AT content, at the same rate observed for the corresponding core genomes [15]. In addition, different environmental conditions and selective pressures [15, 16] may influence the base composition of accessory genes. Selected for stability, core genomes could be more GC rich than accessory genomes due to purifying selection of fitness decreasing or deleterious mutations as mutations predominantly drift towards the more weakly bonded AT base pairs [15, 17]. Selection may also favour homologous recombination of DNA stretches resulting in nucleotide substitutions that are mistaken for single nucleotide polymorphisms (SNPs) [18]. The purpose of the present study was to explore the GC content of the substituted bases (sbGC) in core genomes of strains of diverse microbial species. Each core genome contained at least 10 strains. The substituted bases found in the core genome of each species, ranging from the AT-rich etiologic agent of typhus *Rickettsia prowazekii* (29% GC) to the GC-rich pathogen *Pseudomonas aeruginosa* (66.9% GC), were compared with the corresponding core genome GC content (cgGC). Each of the 35 core genomes represented a unique species from altogether 6 different phyla. We developed a first order differential equation model (gcMOD) predicting sbGC based only on cgGC and fixed parameters estimating (*α*) GC → AT and (*β*) AT→GC substitutions. The parameters *α* and *β* were subsequently estimated using non-linear regression from the empirical data described above and gcMOD.

## Results

**Fig. 1:**
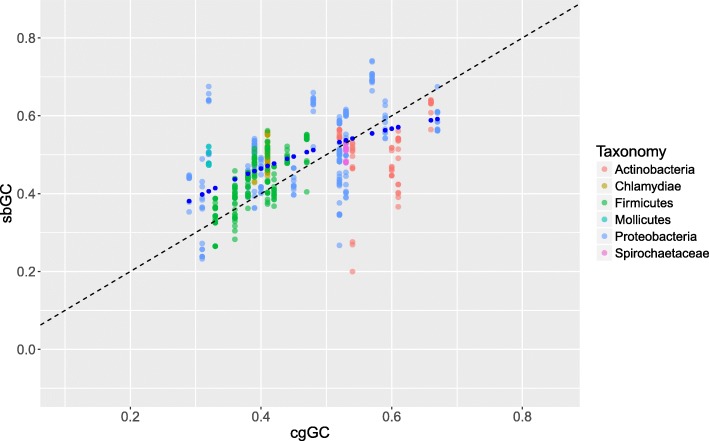
sbGC plotted against cgGC. The graph shows sbGC, for each strain, on the y-axis plotted against corresponding cgGC on the x-axis each point coloured according to phyla. The dashed line designates sbGC = cgGC while the blue points represent gcMOD fitted to the data using non-linear regression

To scrutinize the relationship between sbGC and cgGC we created a simple mathematical model of sbGC. The model (gcMOD) assumes, naively, that sbGC is a function of cgGC, i.e. *F*_*GC*_(*cgGC*)*,* and that sbGC is proportional to AT→GC and GC → AT mutation rates, termed respectively *α* and *β*. Additional file 1 contains a more elaborate mathematical derivation of gcMOD and Additional file 2 gives a more detailed explanation of how sbGC is calculated. Furthermore, gcMOD assumes that the parameters *α* and *β* have different universal fixed rates in the sense that they are not exactly equal (See Additional file 1). To estimate the fixed rates *α* and *β* we employed non-linear least squares regression (nls) with empirical sbGC values, from all strains of each species included in the study, as the response variable against gcMOD, the derived formula for the estimated sbGC function *F*_*GC*_(*x*):

$$ {F}_{GC}(x)=\frac{\beta }{\alpha -\beta}\left({e}^{\left(\alpha -\beta \right)x}-1\right)\kern0.75em (1) $$

where *x* represents cgGC with arbitrary start values set to *α = 2* and *β = 1*. The nls model converged with *σ = 0.076* (residual standard error) suggesting that gcMOD’s sbGC estimates are within ±15.2% (2 *σ* · 100, > 95% Confidence Interval). On convergence, the nls method estimated that *α = (− 0.79 ± 0.25) p < 0.001* and *β = (1.91 ± 0.14) p < 0.001*, which indicates approximately (1.91/0.79) = 2.42 AT substitutions for each GC substitution on average. Figure 1 shows sbGC (vertical axis) for each strain plotted against corresponding cgGC (horizontal axis) together with gcMOD-predicted sbGC values (blue points). Additional files 3 and 4 contains more information regarding the species and data used in the present study. gcMOD also allows for sbGC predictions to be performed for each species, in the sense that each sbGC and cgGC value is based on all strains and all core genomes for each species, if the parameters *α* and *β* are re-estimated accordingly. A similar analysis on bulk sbGC for each core genome (See Additional file 5), i.e. sbGC now designates the bulk %GC content from all strains in each species (each core genome), gives *α = (− 1.35 ± 0.83) p = 0.003* and *β = (2.59 ± 0.61) p < 0.001*, which indicates approximately (2.59/1.35) = 1.92 AT substitutions on average for each GC substitution. With residual standard error *σ = 0.054*, bulk sbGC estimates, which may perhaps be considered as a sort of equilibrium sbGC for each species, are thus within ±11% (2 *σ* · 100, > 95% Confidence Interval).

**Fig. 2:**
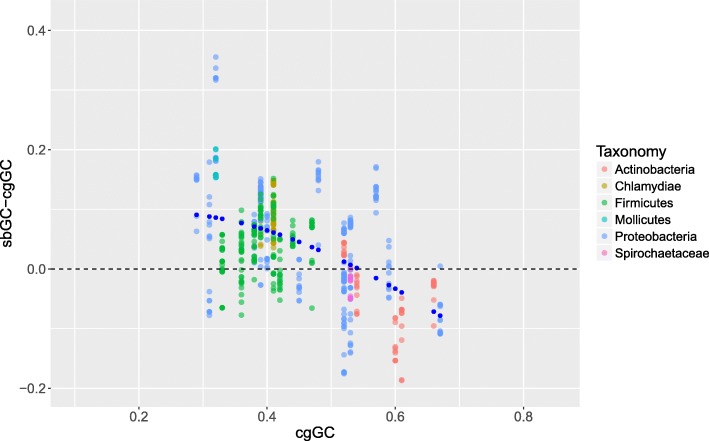
Difference in sbGC- and cgGC plotted against cgGC. The figure shows the difference between sbGC and corresponding cgGC (i.e. sbGC subtracted from cgGC) plotted against cgGC (horizontal axis) as well as the estimated values from (gcMOD-cgGC) (blue points)

It is evident from Fig. 1 that sbGC exhibits a pronounced bias towards higher GC content as compared with cgGC (66% of all strains), assuming that sbGC and cgGC are approximately similar (represented by a dashed line in Fig. 1). Microbes with the highest cgGC (60% or more), however, appear to have more AT-biased substitutions than what would be expected from the trend line (98% of all strains with cgGC> 60%). In other words, the GC-bias of the substituted bases in microbial core genomes appears to level off for the most GC-rich genomes. The GC-rich microbes with AT biased sbGC include mostly Actinobacteria such as *Propionibacterium acnes*, *Mycobacterium tuberculosis, Bifidobacterium longum* and *B. animalis* as well as the γ-proteobacterium *Pseudomonas aeruginosa*. Characteristics shared among these microbes include that they are both free living and host associated (See, for instance, [19]). The magnitude of the GC bias amongst the more AT-rich core genomes is demonstrated in Fig. 2, where cgGC is subtracted from the corresponding sbGC (ΔsbGC) and plotted against cgGC. *F*_*GC*_(*x*) − *x* gives the gcMOD-estimated ΔsbGC and can be seen in Fig. 2 as blue points plotted against the empirical ΔsbGC values. (See Additional file 6 for bulk ΔsbGC for each species/core genome). The figure indicates that there is a negative association between ΔsbGC and cgGC, further emphasizing the observation above that the most AT rich core genomes have, on average, the most GC-biased substituted bases.

In summary, when gcMOD is fitted to empirical sbGC and cgGC values it predicts that there may be, on average, approximately two GC → AT mutations for each AT→GC mutation across the microbial core genomes included in the present study. Since *α* and *β* are fixed parameters the model therefore indicates that mutation rates and the GC → AT bias could be universal for prokaryotes as has been suggested by previous studies [10]. In this respect, the observed sbGC bias for each core genome may be a result of natural selection countering the effects of the universal GC → AT mutation bias [5, 20, 21].

## Discussion

SNP-based phylogeny has fast become the de facto standard for evolutionary analysis of microbial genomes. While recent publications have elucidated a number of mechanisms relating to mutation bias in microbial genomes an examination of mechanisms focusing on the GC content of substituted bases, as well as modelling of AT→GC and GC → AT substitution rates, in microbial core genomes have gained less attention. In the present study, we find a strong non-linear association between sbGC and cgGC where sbGC was more biased towards higher GC content for species with AT-rich core genomes (see Fig. 1). Thus, the most AT-rich species were found to have the most GC-biased substituted bases. sbGC for each species, however, became progressively less biased toward higher %GC with increasing cgGC and, indeed, higher genomic %GC in general. Based on this information we created a mathematical model that demonstrated how sbGC evolves when the respective rates of GC → AT and AT→GC mutations in microbial core genomes vary. Hence, including only a few hypotheses (described in Additional file 1). gcMOD was shown to produce a statistical significant sbGC-biased trend, with regards to cgGC (See Fig. 1 and Additional file 5 for bulk sbGC/cgGC values). That GC → AT outnumber AT→GC mutations in microbial genomes by a significant margin is supported by empirical data [10]. While gcMOD is deterministic, with output taking on a clear curved shape as shown in Fig. 1, empirical sbGC varied between the core genomes of the different species as well as between strains. This has also been widely demonstrated previously as it has been shown how genomic GC content is affected by environment, lifestyle, phylogeny, selective pressures, available molecules such as nitrogen and more [11, 22, 23]. Neither the observation that there are differences regarding energetics between AT and GC nucleotides [24] seem to override the universal trend suggested by gcMOD, at least not for our dataset. Such influences may, however, potentially be manifested in the sbGC variance observed between the strains in each different species (See Fig. 1). While gcMOD estimates that GC → AT mutations may occur, on average, approximately twice as often as AT→GC mutations in microbial genomes the observed biases in the GC content of sbGC, as compared with cgGC, observed in Figs. 1 and 2, are harder to resolve. In fact, the bias in sbGC, as compared with cgGC, is part of an ongoing debate of whether microbial base composition is shaped by natural selection and/or to what extent neutral selective processes may play a significant part [3, 6, 15, 20, 25]. As gcMOD contains some more or less implicit assumptions several consequences can be noted from the observed results. First, sbGC content increments, compared to cgGC, follow a negative trend due to the underlying assumptions of the model. That is, the increment in sbGC substitutions decrease with increasing cgGC (See Fig. 2). This means, for example, that the increase in sbGC for species with cgGC ranging from 50 to 60% GC will, on average, be less than for species with cgGC ranging from 40 to 50% GC. This decreasing trend can also be deduced from Fig. 2 (as well as Additional file 6). Furthermore, and somewhat surprising perhaps, AT → GC / GC → AT mutation biases and rates appear to be remarkably similar for all 35 bacterial species included here, supporting previous observations based on fewer species [10].

It should be noted that selection is less effective on a shorter time scale [16]. Our estimates are however primarily based on different species’ core genomes, consisting of different strains, as opposed to core genomes with intra-clonal isolates separated more recently in time whose mutations may not end up as substitutions or be altogether lacking [10, 16]. Thus, our described results are assumed to be based on the strains of different species of which natural selection (and/or non-selective processes) has had vast opportunities to effectively operate on the different core genomes. It is therefore possible that putative effects of gBGC are concealed by the effects of purifying selection.

While gcMOD appears to give reasonable descriptions of the empirical results, there are some cautions that must be raised. First, the model does not assume anything about the core genomes except that the function estimating sbGC should be a continuously differentiable function of cgGC proportional to mutation rates *α* and *β*. It also means that, on average, the fixed AT/GC substitution rates in each respective core genome follow something that may resemble a universal trend. On average, similar mutation rates for many different species belonging to vastly different phyla, all living in various environments, may seem peculiar. However, statistical significant estimations of the parameters *α* and *β* in gcMOD from empirical sbGC and cgGC values suggests that both similar rates and strongly AT-biased mutations may not be uncommon amongst prokaryotes and this is consistent with previous studies as noted above. Moreover, variable site extraction and removal of recombinant regions are error-prone processes. Indeed, the program Gubbins, used for removal of recombinant sites, is better suited for more closely related isolates with respect to time. Since the substituted bases in each core genome numbered in thousands, errors due to extraction of these substituted bases is expected to be negligible. Furthermore, Gubbins was ran on core genomes of each species, containing both coding and non-coding regions, that are per definition similar with almost identical %GC, together with a core genome-based guide SNP-tree created with the program parSNP. We therefore assume that the variance of sbGC between the strains of the different species is mainly due to life-style, environment and selective pressures. Since we have used data submitted to public databases and these genomes have been sequenced for a reason there could be some kind of implicit selection bias. We have applied strict criteria for inclusion making sure that there are enough strains in each core genome (10 or more) and that the species represent a wide range of genomic GC content found in microbes. Finally, there is obviously also a possibility that our mathematical model is too simple. But its simplicity, we believe, is also its strength as gcMOD significantly fits (*p < 0.001*) the empirical data.

## Conclusions

We present a mathematical model gcMOD that models substituted bases in microbial core genomes (sbGC) each, except for *Brucella* spp., representing a unique species. The model only takes core genome GC content (cgGC) as an independent variable and return estimates of sbGC. gcMOD sbGC predictions are based on parameter estimates of GC → AT and AT→GC substitutions (*β* and *α*, respectively) obtained using non-linear regression on empirical data. We find that sbGC, taken from 35 core genomes, each comprising a unique species, with GC content ranging from 29 to 65% GC, is biased towards higher GC compared with cgGC, for all but the most GC-rich microbial species. This GC-bias could be associated with natural selection to counter the higher GC → AT than AT→GC mutation rates observed universally for microbial genomes. sbGC decreases with respect to increasing cgGC and becomes more AT-biased as core genome %GC increases. It is well known that selection and environmental factors may influence genomic %GC and thus also the sbGC differences between the different species observed here. However, our findings suggest that, given long enough time for selection to operate, the universal higher GC → AT than AT→GC mutation rates may also influence how base composition in microbial core genomes evolves regardless of taxa. Finally, our model support previous observations that the AT-biased mutation rates in bacteria may be pervasive throughout the prokaryotic kingdom and that mutation rates in general are remarkably similar between microbial species from different taxa.

## Methods

All 716 genomes used were taken from a previous study (the archaeon *Sulfolobus islandicus* was removed) and have been thoroughly described there [15]. Briefly, core genomes, containing both coding and non-coding regions, were extracted using parSNP from the Harvest suite tools [26] for prokaryotic species, except *Brucella* spp., having more than 10 fully sequenced strains with closed genomes. It was decided on strict criteria as variant site calling is sensitive to both methods and algorithms used to identify them. Gubbins [18] was subsequently applied on each of the resulting core genomes to remove putative recombinant regions leaving predominantly non-recombinant, substituted bases with a guide tree provided by parSNP for each core genome/species. All Gubbins produced fasta-files were carefully examined using Seaview [27] removing all columns containing gaps. Core genome GC content (cgGC) was both calculated as the total %GC for each core genome, i.e. species containing all strains, but also for each individual strain in each species/core genome. The GC content of the substituted bases (sbGC) was similarly calculated both as the total %GC content of all variable sites for each strain, in each core genome/species, as well as for each individual species. Additional file 2 contains a more elaborate description of how sbGC and cgGC were calculated. Seaview was used to assess %GC in all instances. Both sbGC and cgGC were found to conform to Chargaff’s parity laws [28], i.e. A/T and G/C substitutions are similar.

The derived mathematical model, gcMOD, returns estimated sbGC given cgGC assuming fixed rates *α* and *β* for AT to GC and GC to AT mutation rates. gcMOD is based on the initial condition that sbGC and cgGC are approximately equal as cgGC approaches 0.

To assess model fit, estimate parameters *α* and *β* and determine statistical significance we fitted a non-linear least squares regression model using the function gnls from the R-package “nlme” [29]. Empirical sbGC, from the 35 core genomes described above, was used as the response variable and gcMOD, taking empirical cgGC values, as the non-linear model. The starting values of the parameters were set to *α* = 2 and *β = 1*. The non-linear regression models converged without error and model residuals were assessed and found to follow an approximate Gaussian distribution. The estimated parameters *α* and *β* are reported as mean estimates ± two standard errors (> 95% Confidence Intervals) with a null-hypothesis of being zero (i.e. gcMOD is just a straight horizontal line: $\frac{dF_{GC}(x)}{dx}=0$). All statistical models and figures were created with the free data analysis software R [30]. All figures were created with the R-package ggplot2 [31].

## Additional files

Additional file 1: A detailed mathematical derivation of the sbGC model gcMOD in pdf-format. (PDF 458 kb)

Additional file 2: A detailed explanation of how sbGC and cgGC were derived in pdf-format. (PDF 190 kb)

Additional file 3: An Excel file containing data used for strain-wise sbGC/cgGC analyses (XLSX 44 kb)

Additional file 4: An Excel file containing data used for bulk sbGC/cgGC analyses (XLSX 11 kb)

Additional file 5: The graph shows bulk sbGC on the y-axis plotted against corresponding cgGC on the x-axis for the core genomes of 35 different species each coloured according to phyla. The dashed line designates sbGC = cgGC while the blue points represent gcMOD fitted to the data using non-linear regression. (PDF 8 kb)

Additional file 6: The figure shows the difference between bulk sbGC and corresponding cgGC (i.e. cgGC subtracted from sbGC) plotted against cgGC (horizontal axis) as well as the estimated values from (gcMOD-cgGC) (blue line). (PDF 8 kb)

## Abbrevations

(g)nls: (generalized) non-linear least squares method
AT/GC content: Number of A + T or G + C nucleotides divided by DNA sequence length
cgGC: Core genome GC content
gcMOD: The mathematical model for sbGC prediction
mbp: Mega base pairs; millions of base pairs
sbGC: The GC content of substituted bases in core genomes
SNP: Single nucleotide polymorphism
ΔsbGC: Difference between sbGC and cgGC, i.e. cgGC subtracted from sbGC
