# Supplementary material for: Modeling of the GC content of the substituted bases in bacterial core genomes
Source: BMC Genomics. 2018 Aug 6;19:589. doi: 10.1186/s12864-018-4984-3 (PMC6080486; doi:10.1186/s12864-018-4984-3)
Supplement: Supplementary file 1 — A detailed mathematical derivation of the sbGC model gcMOD in pdf-format. (PDF 458 kb) [file 12864_2018_4984_MOESM1_ESM.pdf]

Below follows a detailed mathematical deduction of gcMOD. We let $F_{GC}(x)$ designate estimated sbGC taking *x*=cgGC as the independent variable. In other words, sbGC is assumed to be a function of cgGC. Furthermore, we assume that

$$F_{GC}(x+\Delta x) = F_{GC}(x) + \alpha\Delta x F_{GC}(x) + \beta\Delta x\big(1 - F_{GC}(x)\big) \quad (1)$$

In other words, as *x*=cgGC increases (or decreases) slightly with *Δx* we assume that $F_{GC}(x+\Delta x)$ will be equal to $F_{GC}(x)$ multiplied with a GC mutation rate parameter α, proportional to the increase in cgGC *Δx*, plus (1-$F_{GC}(x)$), designating estimated sbGC AT content, multiplied by the AT mutation rate parameter β, also proportional to the increased cgGC quantity *Δx*. We thus assume, implicitly, that the increase or decrease in sbGC follows a universal trend governed by the fixed parameters α and β describing the rate of of AT->GC and GC->AT mutations, respectively, as cgGC changes. Phylogenetic relationship will presumably affect sbGC indirectly as it is a function of cgGC meaning that the same functional relation will govern sbGC content in general. The core genomes included are assumed to be at some sort of equilibrium with regards to both sbGC and cgGC. Accepting these hypotheses, we can proceed to rewrite the difference equation (1) as a differential equation by assuming that $F_{GC}(x)$ is a function that has a bounded and continuous derivative:

$$\frac{dF_{GC}(x)}{dx} = \alpha F_{GC}(x) + \beta(1 - F_{GC}(x)) \quad (2)$$

This can be re-written as:

$$\frac{dF_{GC}(x)}{\left(1 + \frac{\alpha-\beta}{\beta}F_{GC}(x)\right)} = \beta dx$$

Using the chain-rule we get:

$$\frac{(\alpha-\beta)dF_{GC}(x)}{\beta\left(1 + \frac{\alpha-\beta}{\beta}F_{GC}(x)\right)} = (\alpha-\beta)dx$$

Resulting in the following relation:

$$d(ln\left(1 + \frac{\alpha-\beta}{\beta}F_{GC}(x)\right)) = (\alpha-\beta)dx$$

Taking the anti-derivatives and letting both sides be the power with respect to the exponential function gives:

$$F_{GC}(x) = \frac{\beta}{(\alpha - \beta)}\left(\mathcal{C}e^{(\alpha-\beta)x} - 1\right) \quad (3)$$

Equation (3) has three parameters that need to be resolved. While α and β will be determined based on empirical data the constant $\mathcal{C}$ is based on some initial condition of the equation. While we assume that $0 < F_{GC}(x) < 1$ we can find $\mathcal{C}$ by letting $\lim_{x \to 0^+} F_{GC}(x) = \epsilon, \epsilon > 0$. Thus, we are assuming that sbGC and cgGC are approximately similar, *i.e.* $F_{GC}(x) \approx x + \epsilon$, as cgGC approaches 0. Due to the assumptions of $F_{GC}(x)$ differences with regards to the initial conditions will be minuscule therefore we can safely assume that $F_{GC}(0) \approx 0$. This initial condition can be considered as tentative and is just to compute a constant that will be fairly representative for $0 < F_{GC}(x) < 1, 0 < x < 1$. The parameters α and β will nevertheless be estimated based on the empirical data. We can then solve for $\mathcal{C}$ which gives the following equation for gcMOD:

$$F_{GC}(x) = \frac{\beta}{(\alpha - \beta)}\left(e^{(\alpha-\beta)x} - 1\right) \quad (4)$$
