# Supplementary material for: Modeling of the GC content of the substituted bases in bacterial core genomes
Source: BMC Genomics. 2018 Aug 6;19:589. doi: 10.1186/s12864-018-4984-3 (PMC6080486; doi:10.1186/s12864-018-4984-3)
Supplement: Supplementary file 2 — A detailed explanation of how sbGC and cgGC were derived in pdf-format. (PDF 190 kb) [file 12864_2018_4984_MOESM2_ESM.pdf]

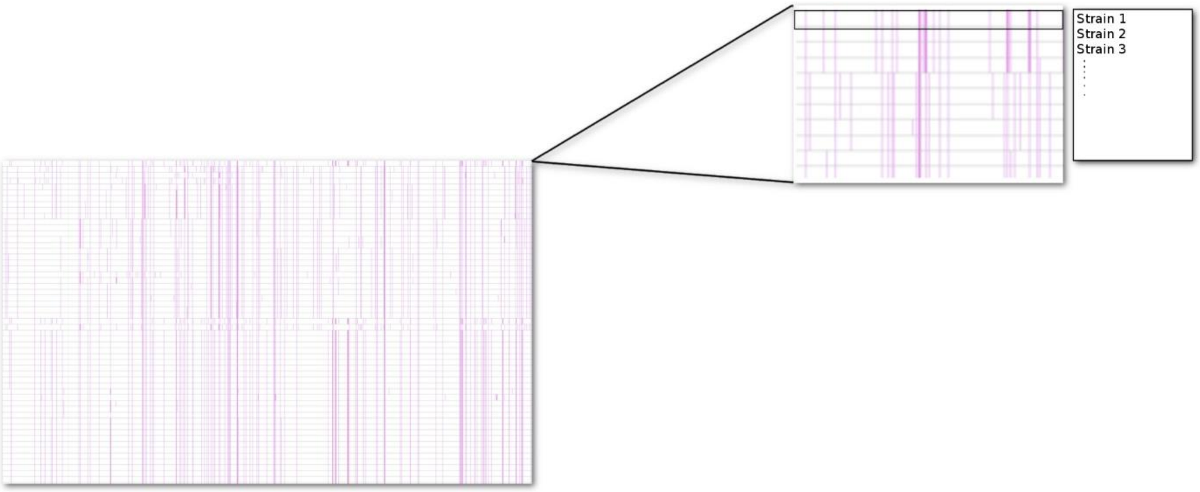

Extraction of substituted bases and estimation of their GC content in microbial core genomes. The core genomes consist of coding and non-coding regions similar to all strains (at least 10) in a species (excepting *Brucella* spp.). Harvesttools/parSNP [1] was used to extract the core genomes. The substituted bases in the core genomes were extracted by removing all bases identical to all strains. This can be illustrated with the figure above which depicts an extract from a microbial core genome (*Chlamdydia trachomatis*). Each vertical row (see extract to the top right) represents one strain of *C. trachomatis*. The vertical lines within each row colored white represents similar bases with the other strains while the pink lines represent substituted bases. Both the white and pink lines can be any base A,G,C and T. To calculate the GC content of the substituted bases (sbGC) of each strain (78 for *C. trachomatis*) only all white lines that run through all strains (see panel to the left above) in a species are removed with Gubbins [2] (together with putative recombined bases) meaning that each strain will have the same number of substituted bases (3760 bp for each strain of *C. trachomatis*). The remaining bases in each row are used to calculate sbGC for each strain. All white lines removed from all strains (1026916 bp for *C. trachomatis*) are used to calculate the core genome %GC. Strains with few substituted bases will presumably have sbGC similar to core genome %GC. Bulk sbGC is taken to mean the total %GC of substituted bases for all strains in a species.

[1] Treangen TJ, Ondov BD, Koren S, Phillippy AM: The Harvest suite for rapid core-genome alignment and visualization of thousands of intraspecific microbial genomes. Genome Biol 2014, 15(11):524.

[2] Croucher NJ, Page AJ, Connor TR, Delaney AJ, Keane JA, Bentley SD, Parkhill J, Harris SR: Rapid phylogenetic analysis of large samples of recombinant bacterial whole genome sequences using Gubbins. Nucleic Acids Res 2015, 43(3):e15.
