# Supplementary figures and images for: Modeling of the GC content of the substituted bases in bacterial core genomes
Source: BMC Genomics. 2018 Aug 6;19:589. doi: 10.1186/s12864-018-4984-3 (PMC6080486; doi:10.1186/s12864-018-4984-3)

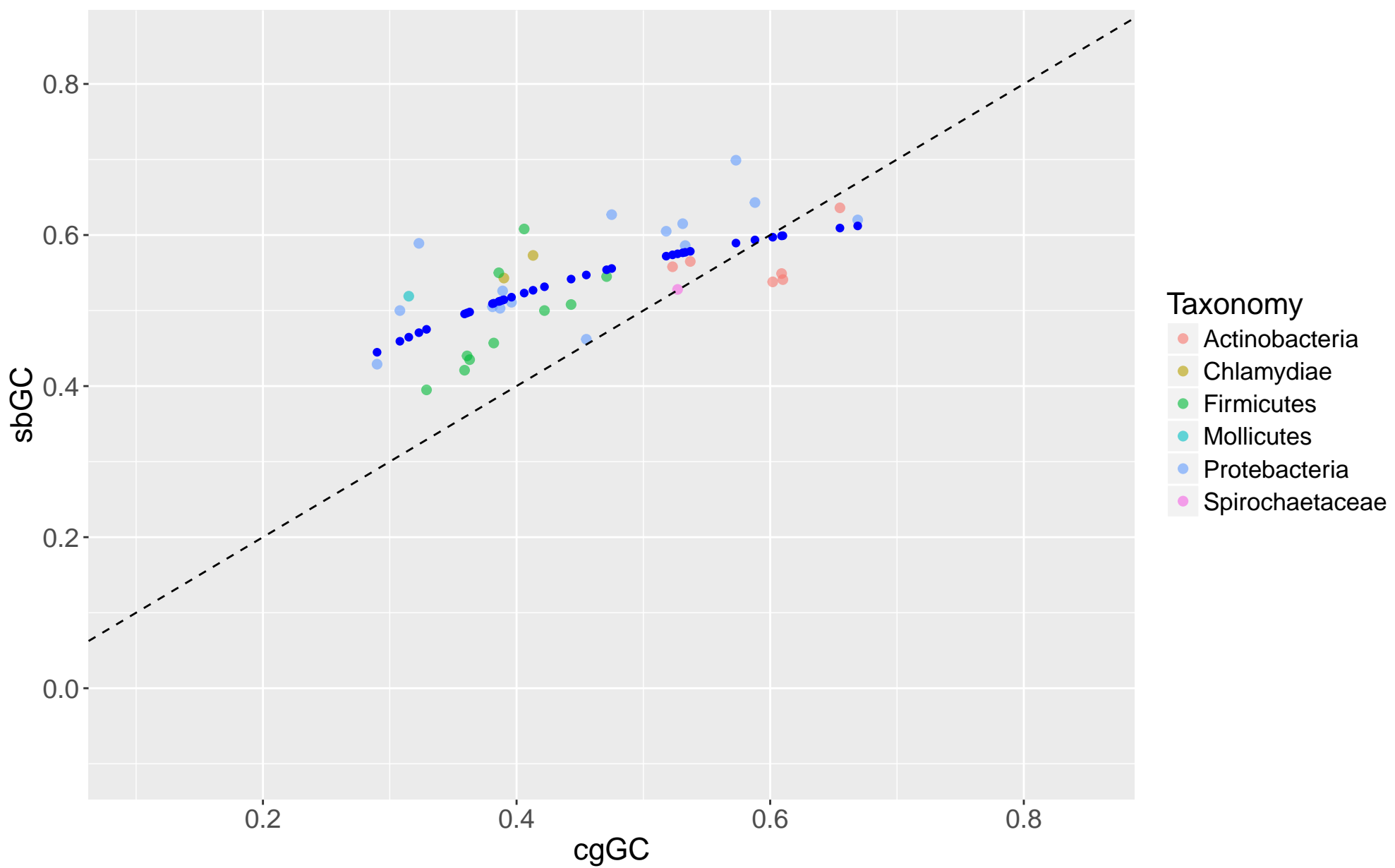

Supplement: Supplementary file 5 — The graph shows bulk sbGC on the y-axis plotted against corresponding cgGC on the x-axis for the core genomes of 35 different species each coloured according to phyla. The dashed line designates sbGC = cgGC while the blue points represent gcMOD fitted to the data using non-linear regression. (PDF 8 kb) [file 12864_2018_4984_MOESM5_ESM.pdf]

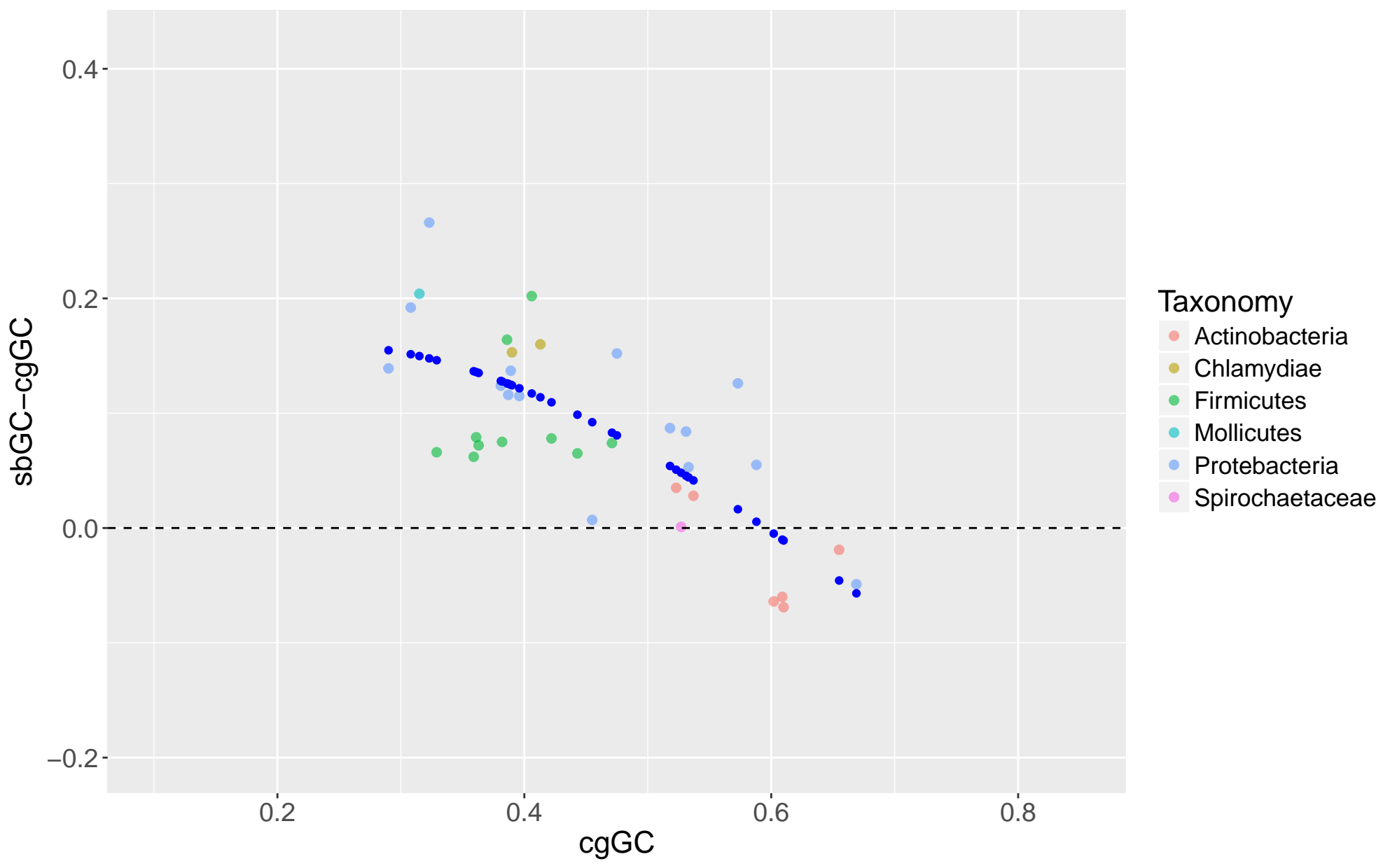

Supplement: Supplementary file 6 — The figure shows the difference between bulk sbGC and corresponding cgGC (i.e. cgGC subtracted from sbGC) plotted against cgGC (horizontal axis) as well as the estimated values from (gcMOD-cgGC) (blue line). (PDF 8 kb) [file 12864_2018_4984_MOESM6_ESM.pdf]
